# Threshold Validity of N3 Criteria in Nasopharyngeal Carcinoma: A Narrative Methodological Review of Pragmatic Cutoffs and Biologically Defensible Risk Boundaries

**DOI:** 10.3390/clinpract16080156

**Published:** 2026-08-21

**Authors:** Erkan Topkan, Efsun Somay, Melis Selek, Ugur Selek

**Affiliations:** 1 Department of Radiation Oncology, School of Medicine, Başkent University, Ankara 06754, Türkiye; docdretopkan@gmail.com; 2 Department of Oral and Maxillofacial Surgery, Faculty of Dentistry, Başkent University, Ankara 06754, Türkiye; 3 School of Medicine, Koc University, Istanbul 34340, Türkiye; mselek22@ku.edu.tr; 4 Department of Radiation Oncology, School of Medicine, Koç University, Istanbul 34340, Türkiye; ugurselek@yahoo.com

**Keywords:** nasopharyngeal carcinoma, N3 staging, threshold validity, nodal volume, radiologic extranodal extension, continuous-risk modeling

## Abstract

This narrative methodological review aims to critically assess whether current AJCC/UICC N3-defining criteria for nasopharyngeal carcinoma (NPC) represent validated biological and statistical thresholds or pragmatic staging boundaries. Specifically, it examines the evidential basis of the >6 cm nodal-size threshold, extension below the caudal border of the cricoid cartilage, and advanced radiologic extranodal extension (rENE), with emphasis on the distinction between prognostic-variable validity and threshold validity. The >6 cm criterion is a historically inherited threshold that predates contemporary MRI-based staging and may insufficiently capture the three-dimensional complexity of nodal tumor burden. Similarly, defining inferior nodal extension by the caudal border of the cricoid cartilage improves anatomical reproducibility compared with earlier lower-neck definitions, yet it remains a pragmatic imaging landmark rather than a validated biological boundary for lymphatic dissemination. By contrast, advanced rENE is more biologically informative because it reflects invasive tumor behavior; nonetheless, its staging utility depends on standardized imaging definitions, interobserver reliability, external validation, and incremental clinical utility. Emerging imaging-derived descriptors—including MRI-based nodal diameter, nodal volume, middle-neck involvement, total tumor volume, and integrated imaging–biological models—underscore the limitations of relying exclusively on single categorical thresholds. Future N3 refinement should move beyond substituting one cutoff for another by characterizing nodal descriptors in continuous, ordinal, volumetric, or severity-graded forms before adopting simplified categories. Future refinement of NPC nodal staging will likely require approaches that move beyond isolated anatomical thresholds and better account for the multidimensional nature of nodal disease, including tumor burden, spatial distribution, invasive characteristics, and biological risk.

## 1. Introduction

Nasopharyngeal carcinoma (NPC) is characterized by a distinct epidemiological distribution, close association with Epstein–Barr virus, marked radiosensitivity, and a characteristic pattern of lymphatic dissemination [1]. Cervical nodal involvement is common at diagnosis and, because it is strongly associated with distant metastasis, represents a major determinant of prognosis, treatment planning, and surveillance [1,2]. Accordingly, N classification is central to prognostic grouping, clinical communication, trial stratification, and decisions regarding systemic treatment intensity [2,3]. In clinical practice, patients with N3 disease generally receive definitive concurrent chemoradiotherapy, while those with particularly high-risk nodal burden may be considered for treatment intensification, including induction chemotherapy followed by concurrent chemoradiotherapy. Accurate identification of advanced nodal disease is therefore relevant not only to prognostic stratification but also to treatment selection, surveillance planning, and patient counseling. Future refinements of N classification should ideally improve both prognostic discrimination and clinical decision-making without compromising reproducibility or practicality. Although nodal involvement contributes substantially to overall clinical stage, N-positive disease does not correspond to a single stage group. Overall stage in NPC is determined by the combined T, N, and M categories; consequently, patients with similar nodal status may be assigned to different clinical stages depending on the extent of the primary tumor and the presence or absence of distant metastasis. With the widespread use of magnetic resonance imaging (MRI) and intensity-modulated radiotherapy, nodal assessment has become increasingly precise, encompassing size, laterality, retropharyngeal involvement, inferior neck extension, nodal volume, extranodal extension, and spatial distribution [3,4,5,6]. However, whether current categorical N-stage thresholds adequately capture this enhanced anatomical and biological resolution remains uncertain.

Among the N categories, N3 disease identifies the highest nodal-risk burden in non-metastatic NPC and is associated with increased distant failure and inferior survival [2,3]. In current AJCC/UICC staging frameworks, N3 classification is defined by large nodal size—classically, a lymph node exceeding 6 cm—and/or inferior extension below the caudal border of the cricoid cartilage, with recent revisions also incorporating advanced radiologic extranodal extension (rENE) [7]. Although these criteria provide pragmatic and reproducible categories for routine clinical use, they transform complex, continuous, and spatially distributed nodal characteristics into discrete staging boundaries.

Prognostic relevance, however, does not establish the validity of a particular cutoff or anatomical boundary. Nodal size and volume are continuous variables, whereas inferior nodal extent is at least ordinal and potentially spatially continuous. Thus, patients immediately above and below a staging boundary—for example, with nodal diameters of 6.1 and 5.9 cm—are unlikely to differ fundamentally in disease biology solely because of that categorization. This does not negate the clinical utility of staging systems, but underscores the distinction between pragmatic classification and threshold validity [8,9].

The traditional >6 cm criterion illustrates this concern. Although larger nodal size plausibly reflects greater tumor burden and adverse prognosis, the 6 cm threshold was inherited from earlier staging conventions and has remained within the UICC/AJCC N3 definition since the fourth edition in 1992 [4]. It was not derived from contemporary MRI-based continuous-risk modeling, and imaging-era studies have questioned its prognostic adequacy. Huang et al., for example, evaluated MRI-determined nodal size in intensity*modulated radiotherapy (IMRT)-treated NPC and identified maximal axial diameter, with 4 cm as a candidate threshold, as a potentially more relevant descriptor [5]. Similarly, although the caudal border of the cricoid cartilage is a convenient landmark for lower-neck extension, evidence that it represents a biological boundary of lymphatic dissemination remains limited. Inferior nodal extent may therefore be prognostically relevant even if the cricoid border is not the optimal dichotomous threshold.

Recent large-scale staging-validation studies have reinforced the prognostic relevance of selected nodal features [3,7]. Nevertheless, statistical significance does not establish that existing thresholds represent biological inflection points or outperform continuous nodal size, nodal volume, nodal-level burden, distance-based inferior extent, rENE grading, or integrated biomarker-imaging models [5,6,8,9,10,11]. This is particularly important in very large cohorts, in which modest associations may achieve statistical significance without clinically meaningful improvements in discrimination, calibration, reclassification, or decision utility [9,10,11].

Accordingly, the principal aim of this review is not to dispute the clinical utility of the current AJCC/UICC N-staging system or merely to summarize emerging prognostic markers, but to critically appraise whether the thresholds defining N3 disease and the proposed imaging-derived and biological alternatives demonstrate sufficient methodological robustness, reproducibility, and incremental clinical utility to support future staging refinement. Particular attention is given to the historical >6 cm nodal-size criterion and the caudal border of the cricoid cartilage as pragmatic boundaries whose biological and statistical foundations merit reassessment, together with candidate descriptors including MRI-derived nodal diameter, nodal volume, extranodal extension, middle-neck involvement, and integrated imaging–biological models. Because N3 disease is closely linked to the risk of distant failure, improved risk stratification could enable more precise identification of patients most likely to benefit from induction chemotherapy or intensified systemic therapy, tailored surveillance strategies, and more appropriate clinical trial stratification. Even modest improvements in risk discrimination could therefore be clinically meaningful while also providing an evidence-based framework for the future evolution of N-stage classification [5,6,8,9,10,11].

**Figure 1:**
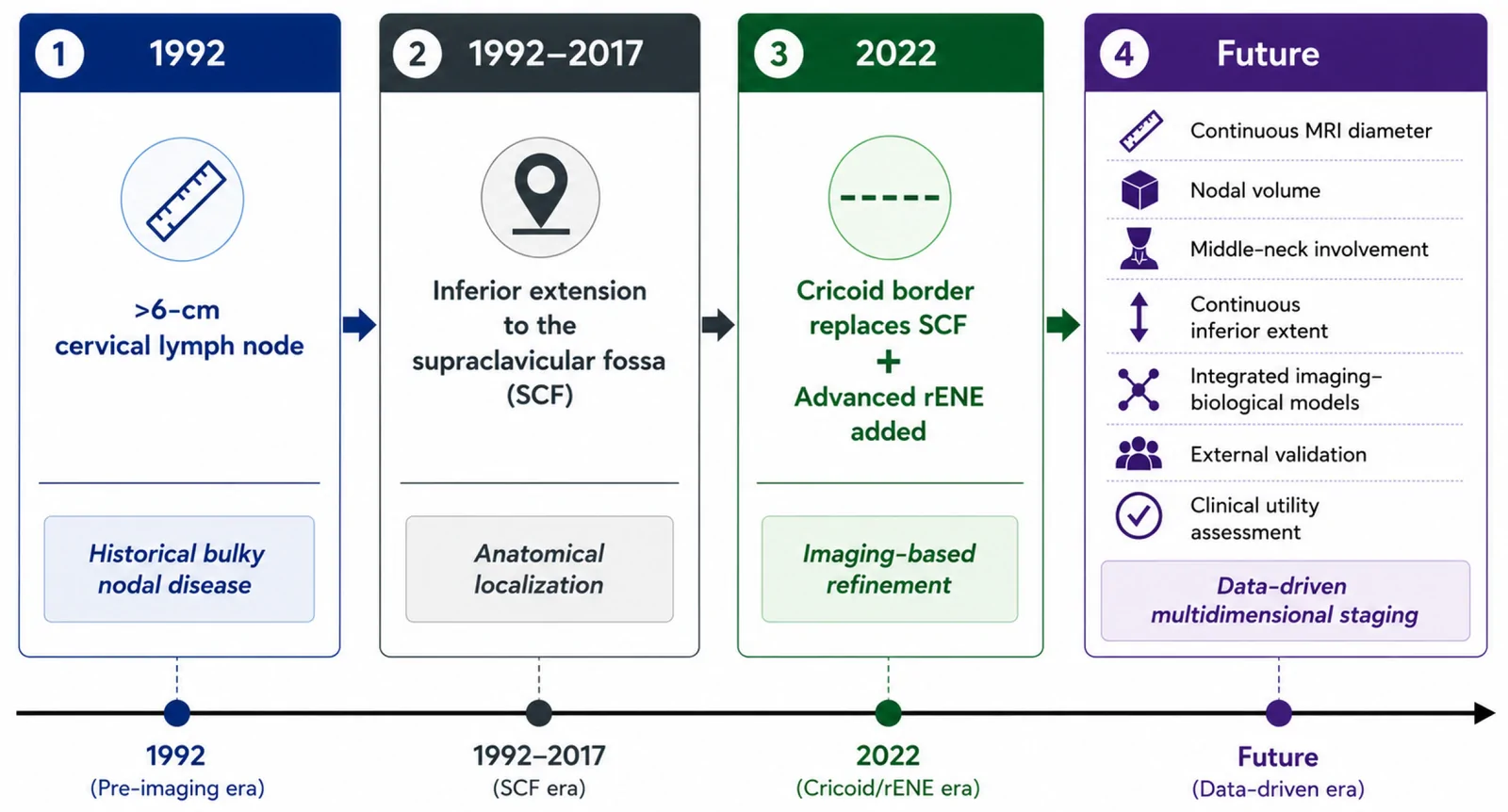
Evolution of the AJCC/UICC N3 classification in nasopharyngeal carcinoma and future directions for staging refinement. The N3 category has evolved from a predominantly size-based definition centered on a cervical lymph node diameter exceeding 6 cm to anatomically defined inferior nodal extension involving the supraclavicular fossa. In the ninth AJCC/UICC edition, the supraclavicular fossa criterion was replaced by extension to or below the caudal border of the cricoid cartilage, and advanced radiologic extranodal extension was incorporated as an additional N3-defining feature. Future refinement may increasingly rely on continuous MRI-derived nodal diameter, nodal volume, middle-neck involvement, continuous inferior nodal extent, integrated imaging–biological models, external validation, and demonstration of incremental clinical utility. Abbreviations: AJCC, American Joint Committee on Cancer; UICC, Union for International Cancer Control; SCF, supraclavicular fossa; rENE, radiologic extranodal extension; MRI, magnetic resonance imaging.

Importantly, this review does not advocate the immediate replacement of the current AJCC/UICC N3 criteria with MRI-based nodal diameter, nodal volume, middle-neck involvement, or integrated imaging–biological models. Although these candidate descriptors may provide valuable prognostic information and warrant further validation, the available evidence does not establish any specific alternative cutoff or anatomical boundary as superior to the current N3 criteria or sufficiently validated for routine clinical staging. Rather, these descriptors should be regarded as promising candidates against which the current criteria may be benchmarked through continuous-risk characterization, reproducibility assessment, external validation, and systematic evaluation of incremental clinical utility (Figure 1).

## 2. Review Design and Literature Selection

This article was designed as a narrative methodological review rather than a systematic review or meta-analysis. The objective was not to generate pooled effect estimates, but to critically examine the evidential and methodological basis of current and emerging N3-defining criteria in NPC, with particular emphasis on threshold validity, continuous-risk characterization, imaging-derived nodal descriptors, and clinical utility.

The relevant literature was identified through targeted searches of PubMed, Web of Science, and Google Scholar, supplemented by citation tracking from key staging and imaging studies. Searches were conducted up to May 2026 using combinations of terms including “nasopharyngeal carcinoma,” “N3 staging,” “AJCC/UICC,” “nodal size,” “nodal volume,” “cricoid cartilage,” “lower neck involvement,” “radiologic extranodal extension,” “EBV DNA,” “threshold validation,” “dichotomization,” “restricted cubic splines,” “calibration,” “external validation,” and “decision-curve analysis.” Priority was given to studies addressing AJCC/UICC nasopharyngeal carcinoma staging, N-classification refinement, MRI-based nodal assessment, nodal size, nodal volume, inferior nodal extent, rENE, EBV DNA, and methodological issues related to dichotomization, prediction modeling, calibration, validation, and clinical utility.

Studies were selected for inclusion based on relevance to the review’s central question: whether current and proposed N3-related descriptors represent prognostically useful variables, statistically valid thresholds, or pragmatic staging boundaries. Because this was a narrative review, no formal risk-of-bias assessment, pooled analysis, or certainty-of-evidence grading was performed. This design was chosen to allow conceptual synthesis across staging, imaging, and methodological literature.

## 3. Current N3 Definition and Historical Evolution

The conceptualization of advanced nodal disease in NPC has undergone progressive refinement in parallel with advances in imaging, radiotherapy, and outcome modeling. Initial staging systems predominantly relied on clinical examination, palpation-based nodal assessment, and broad anatomical descriptors. The advent of MRI-based staging and IMRT has enabled substantially greater anatomical precision, leading to successive updates of the AJCC/UICC N classification [3,4]. Despite these advances, several foundational aspects of the N3 definition—especially the criteria for large nodal size and inferior neck extension—have remained anchored in earlier staging paradigms. The historical evolution of the AJCC/UICC N3 classification and the anticipated trajectory of future staging refinement are summarized in Figure 1.

Within the current AJCC/UICC staging, N3 disease designates the most advanced nodal category in non-metastatic NPC. The prevailing definition encompasses cervical lymph node metastases with a greatest dimension exceeding 6 cm and/or extension below the caudal border of the cricoid cartilage, while the ninth edition further incorporates advanced rENE as an N3-defining feature [7]. This framework reflects a longstanding premise that extensive nodal burden, inferior nodal spread, and aggressive extranodal behavior delineate a subgroup at increased risk for adverse outcomes, most notably distant failure and reduced survival [3,7].

The >6 cm nodal-size criterion remains among the most enduring features of advanced nodal staging. Historically, this threshold was introduced in the UICC/AJCC NPC N3 subset in the fourth edition in 1992 and has been retained in subsequent iterations [4]. Its persistence may be attributable to both historical continuity and clinical convenience, as a single unidimensional size cutoff is straightforward to implement, communicate, and integrate into staging algorithms. Notably, the establishment of the 6 cm threshold predates routine MRI-based nodal assessment and was not derived from modern continuous-risk modeling of nodal diameter. Thus, while substantial nodal size is clinically plausible as an indicator of elevated tumor burden, the specific value of 6 cm is more accurately regarded as a pragmatic staging demarcation rather than a biologically validated inflection point [4,5].

The criterion for inferior-neck involvement has experienced a more anatomically explicit evolution. Earlier staging systems used supraclavicular fossa involvement to define advanced nodal disease; however, the anatomical boundaries of the supraclavicular fossa have proven difficult to delineate consistently, especially on imaging. In the context of IMRT-era advancements, Pan et al. advocated, in the eighth edition, replacing the supraclavicular fossa criterion with an extension below the caudal border of the cricoid cartilage—a landmark considered more reproducible on imaging [3]. While this modification enhanced operational clarity, it also rendered inferior nodal extent a binary anatomical parameter, classifying nodal disease based on traversal of a specific horizontal plane.

The ninth edition of the AJCC/UICC framework maintained the central emphasis on inferior nodal extension and further refined the N3 category by incorporating advanced rENE [7]. This addition holds conceptual significance, as extranodal extension reflects invasive tumor behavior rather than nodal size or anatomical position alone. In this context, advanced rENE may serve as a more biologically informative marker of aggressive nodal disease compared to purely anatomical thresholds. Nevertheless, its inclusion also underscores the broader trajectory of NPC staging, which is shifting from straightforward anatomical classification toward the integration of imaging-defined biological risk descriptors [7].

Despite these refinements, the current N3 definition remains a composite of historical, anatomical, and imaging-derived criteria. The >6 cm criterion retains a longstanding size-based convention; the caudal border of the cricoid cartilage offers a reproducible anatomical proxy for lower-neck involvement; and advanced rENE introduces a more behaviorally oriented imaging marker [3,4,7]. While this hybrid structure is clinically practical, it also raises a central methodological consideration: have the thresholds delineating N3 disease been validated as optimal representations of continuous nodal risk, or have they been perpetuated primarily because of their simplicity, reproducibility, and alignment with earlier staging paradigms?

This distinction is pivotal when interpreting current N3 staging. Evidence supporting nodal size, inferior extension, and extranodal extension as adverse prognostic variables does not, in itself, validate the specific categorical boundaries established to define them. A given variable may possess prognostic relevance, yet the designated threshold may lack robust justification. Accordingly, the historical evolution of N3 staging should be viewed not only as a series of refinements in anatomical specification but also as an illustration of the ongoing balance among prognostic association, clinical utility, reproducibility, and continuity with prior classifications. While this balance is essential for global staging systems, it should not preclude periodic reassessment of whether established thresholds remain optimal in the context of MRI- and IMRT-era practice.

## 4. Variable Validity Versus Threshold Validity

**Figure 2:**
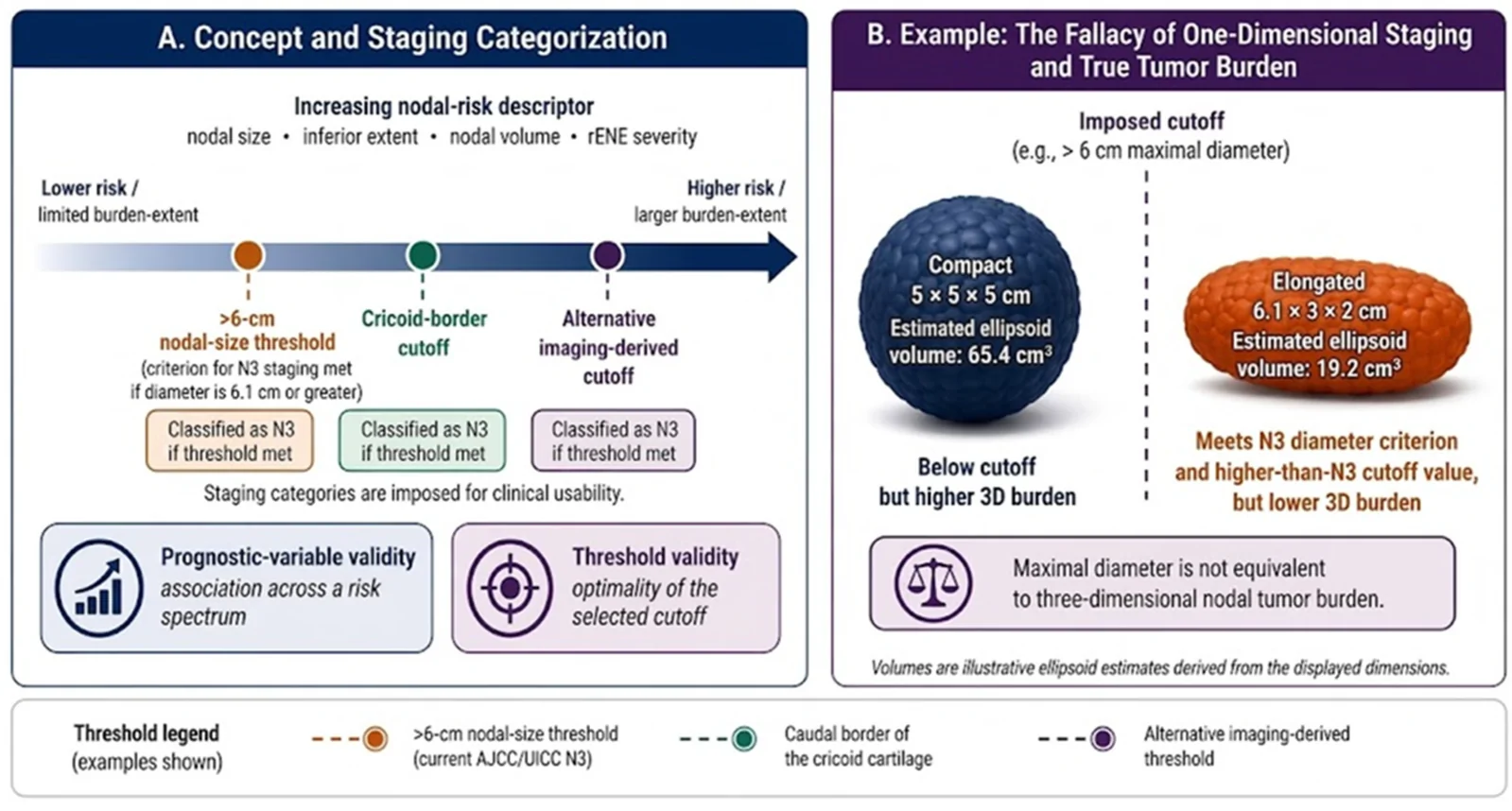
Conceptual distinction between prognostic-variable validity and threshold validity in nasopharyngeal carcinoma N3 staging. Note: This schematic does not represent patient-level outcome data or a fitted statistical model. Panel (**A**) illustrates that nodal-risk descriptors may carry prognostic information across a continuous or ordinal spectrum, whereas staging systems impose categorical thresholds for clinical usability. Panel (**B**) illustrates how a unidimensional diameter threshold may conflict with three-dimensional nodal tumor burden. The displayed volumes are illustrative geometric and ellipsoid estimates derived from the shown dimensions and do not represent imaging-segmented nodal volumes. Prognostic association of a descriptor does not, by itself, establish that a specific cutoff represents a biological or statistical inflection point. Abbreviations: AJCC, American Joint Committee on Cancer; NPC, nasopharyngeal carcinoma; rENE, radiologic extranodal extension; UICC, Union for International Cancer Control.

A fundamental methodological consideration in staging research concerns the distinction between prognostic-variable validity and threshold validity. Prognostic-variable validity addresses whether a clinical, anatomical, imaging, or biological feature is reproducibly associated with a given outcome. In contrast, threshold validity concerns whether the cutoff or boundary used to categorize that feature constitutes an optimal, reproducible, and clinically meaningful point along the risk continuum. Although these constructs are related, they are not synonymous: a variable may exhibit strong prognostic relevance, while the corresponding threshold selected for dichotomization may remain statistically unstable, biologically unsubstantiated, or clinically suboptimal [8,9,12]. This conceptual distinction between prognostic-variable validity and threshold validity is illustrated schematically in Figure 2.

This distinction is particularly relevant in the context of NPC nodal staging. Features such as nodal size, inferior nodal extent, nodal volume, and extranodal extension may each possess prognostic significance. Nevertheless, evidence that these variables are associated with survival, distant failure, or regional control does not, in itself, validate the specific staging boundaries chosen to categorize them [8,9,10,12]. The observation that larger nodes are associated with a poorer prognosis does not, for instance, establish 6 cm as the optimal size threshold. Similarly, the association between inferior nodal spread and adverse outcomes does not confirm the caudal border of the cricoid cartilage as the most appropriate anatomical delineation for N3 disease. In both scenarios, the prognostic variable and its categorical threshold warrant separate validation processes.

The methodological challenge arises because several nodal descriptors are inherently continuous or ordinal. Nodal diameter and nodal volume are measured along continuous scales, while inferior nodal extension reflects a spatial continuum along the cervical axis rather than a binary anatomical state. While dichotomization of such variables may offer practical advantages for staging, it can also result in the loss of prognostic information, diminished statistical power, the introduction of imposed risk discontinuities, and the potential misrepresentation of nonlinear risk associations. Furthermore, this approach may inadvertently amplify the perceived significance of cases situated in close proximity to the imposed cutoff [8,9,12]. Consequently, patients whose nodal disease lies just below a given threshold may be assigned to a different N category than those just above it, despite likely having comparable underlying biological risk.

This limitation is not unique to NPC staging. The broader statistical literature has consistently cautioned against dichotomizing continuous predictors, noting that such practices may lead to information loss, residual confounding, diminished model performance, and potentially misleading interpretations of risk relationships [8,12]. In prognostic modeling, it is generally preferable to preserve continuous variables whenever feasible and to investigate potential nonlinear associations using flexible modeling strategies before proposing simplified thresholds [9,10]. Where categorization is deemed necessary for clinical applicability, the chosen cutoff should ideally be substantiated by evidence indicating that it reflects a meaningful inflection in risk, rather than simply yielding statistically significant group distinctions [9,10,11].

Within the context of N3 staging, the pertinent inquiry extends beyond a given feature’s prognostic capacity. The central consideration is whether the selected boundary most accurately reflects the underlying risk gradient. For the >6 cm criterion, the relevant question is whether risk changes meaningfully near the 6 cm threshold, or whether nodal diameter is better conceptualized as a continuous, nonlinear, or volumetric variable [5,6,8,9,10,12]. Regarding the caudal border of the cricoid cartilage, it is necessary to ascertain whether inferior nodal extent exhibits a discernible risk inflection at this anatomical landmark, or if alternative constructs—such as nodal level, distance-based inferior extent, number of involved nodal levels, or region-specific nodal volume—might more effectively represent risk [3,7,9,10]. For advanced rENE, the considerations differ somewhat: while this descriptor has plausible biological relevance as an indicator of invasive tumor behavior, its utility in staging remains contingent upon reproducible imaging criteria, interobserver consistency, and demonstrable prognostic value beyond existing N-category features [7,9,10,11].

A methodologically robust approach to threshold validation should proceed in a systematic sequence. Initially, the variable of interest should be analyzed in its continuous or ordinal form. Subsequently, the relationship between the variable and the outcome should be explored using flexible modeling techniques, such as restricted cubic splines, fractional polynomials, or penalized spline-based Cox regression methods [9,10]. Third, it is important to assess nonlinearity and identify genuine inflection points before selecting a simplified threshold. Should a cutoff be proposed, its predictive performance ought to be evaluated through internal validation—ideally utilizing bootstrapping or cross-validation—and subsequently corroborated in independent external cohorts [9,10]. Finally, the proposed threshold should be benchmarked against alternative representations by assessing discrimination, calibration, reclassification, and clinical utility metrics, including decision-curve analysis when indicated [9,10,11].

This framework underscores a fundamental limitation of many staging refinements. Analyses involving large cohorts may reveal that a binary feature is statistically associated with the outcome; however, this does not inherently establish the optimality of the binary threshold employed [9,10,12]. Statistical significance may signal that a variable is prognostically informative, yet it does not guarantee that the chosen cutoff most accurately or usefully reflects the risk relationship. This distinction is especially salient in the context of very large datasets, where modest differences may achieve statistical significance without yielding clinically meaningful advances in discrimination, calibration, or decision utility [10,11].

Accordingly, future refinements of NPC N staging would benefit from explicitly distinguishing between validating prognostic variables and validating thresholds. The most methodologically robust strategy may involve characterizing nodal burden and inferior nodal extent as continuous or ordinal variables, assessing for genuine risk inflection points, and subsequently translating these findings into simplified categories only when such categorization enhances clinical interpretability without incurring undue loss of prognostic information [8,9,10,11,12]. This conceptual distinction underpins the rationale for re-examining the >6 cm nodal-size criterion, the caudal border of the cricoid cartilage, and emerging imaging-derived alternatives in subsequent sections.

## 5. The >6 cm Criterion: Historical Continuity, Unidimensional Measurement, and Volumetric Discordance

The >6 cm nodal-size criterion remains one of the most enduring elements of advanced nodal staging in NPC. Its incorporation into the UICC/AJCC N3 subset in the fourth edition (1992) provided a straightforward, clinically accessible approach for identifying patients with bulky cervical nodal disease [4]. The historical appeal for this criterion is apparent: a unidimensional threshold is readily measured, communicated, and implemented across diverse clinical settings. Nevertheless, the persistence of this cutoff should not be conflated with evidence that the 6 cm boundary constitutes a biologically validated or statistically optimal demarcation of risk. The key issue is not merely whether nodal size is prognostically relevant, but whether 6 cm represents the most appropriate threshold for categorical reclassification as N3.

Several factors constrain the current evidential support for the >6 cm criterion. Importantly, this threshold was established prior to the widespread adoption of MRI-based nodal assessment and thus reflects an era in which clinical examination and palpation predominated [4]. More recent evidence from the MRI era has called into question the continued relevance of the 6 cm boundary as the most informative size descriptor. For example, Huang et al. examined MRI-determined cervical lymph node size in IMRT-treated NPC and identified a maximal axial diameter of 4 cm as a potential prognostic threshold for future refinement [5]. In parallel, Ni et al. found that maximum cervical lymph node diameter did not independently predict prognosis in locoregionally advanced NPC treated with IMRT, further implying that unidimensional nodal size alone may be insufficient to capture risk in contemporary practice [13].

A key conceptual limitation of the >6 cm criterion lies in its reliance on a single linear measurement. Nodal disease is inherently three-dimensional, frequently irregular, and may present as matted or necrotic, with involvement spanning multiple nodal levels. This concern is consistent with radiologic observations that malignant cervical nodes often exhibit altered morphology, including a more rounded configuration and changes in the short-to-long axis ratio, rather than being adequately characterized by a single maximum diameter [14]. As such, the maximum diameter may not consistently reflect true nodal tumor burden. For instance, an elongated nodal mass measuring 6.1 × 3 × 2 cm yields a rectangular box-volume approximation of 36.6 cm^3^ and an estimated ellipsoid volume of 19.2 cm^3^ [(π/6) × 6.1 × 3 × 2]. In contrast, a more compact nodal mass measuring 5 × 5 × 5 cm yields a rectangular box-volume approximation of 125 cm^3^ and an estimated ellipsoid volume of 65.4 cm^3^ [(π/6) × 5 × 5 × 5]. Thus, even when a more anatomically plausible ellipsoid approximation is applied, a compact node below the 6 cm threshold may retain substantially greater estimated volume than an elongated node that just exceeds the unidimensional size criterion, underscoring the limitations of diameter-based staging rules in capturing biologically relevant nodal burden.

This volumetric discordance is clinically significant, as multiple studies suggest that tumor burden in NPC may be better represented by volumetric measures than by unidimensional diameter. Yuan et al. assessed quantitative MRI and PET/CT parameters for NPC risk stratification and found that cervical nodal volume was prognostically informative, serving as a high-risk descriptor in their model [6]. Similarly, Chen et al., in a cohort of 1230 patients with cervical nodal metastases, demonstrated that cervical nodal tumor volume was associated with treatment outcomes, particularly regional control [15]. Liang et al. also found that total tumor volume, encompassing both primary and nodal tumor burden, was prognostically significant in IMRT-treated NPC [16]. Collectively, these data support the proposition that three-dimensional tumor burden may better encapsulate biologically meaningful risk than a single maximum diameter.

However, identifying alternative size or volume metrics does not inherently justify substituting one threshold for another. The methodological standards applied to the 6 cm cutoff are equally applicable to any proposed MRI-based diameter or volumetric thresholds. For example, a 4 cm maximum axial diameter or an 18-cc nodal volume threshold should not be regarded as definitive solely on the basis of survival curve separation or statistical significance within a specific cohort. Rather, such thresholds warrant comprehensive continuous-risk modeling, assessment of potential nonlinearity, identification of genuine inflection points, and validation in independent datasets [8,9,10,12]. The principal objective should be to ascertain whether a candidate cutoff approximates a reproducible risk inflection, rather than simply generating statistically distinct groups.

The >6 cm criterion, therefore, exemplifies the broader problem of threshold inheritance in staging systems. Once a cutoff becomes embedded in an official classification, it may persist because it offers continuity, simplicity, and global reproducibility. These are valuable properties for clinical staging, but they do not substitute for contemporary threshold validation. In the MRI and IMRT era, it is reasonable to reassess whether a historical unidimensional cutoff remains the best representation of nodal risk when more detailed information is available, including nodal volume, number of involved nodes, nodal-level distribution, inferior extent, extranodal extension, and circulating EBV DNA, where available.

This discussion does not suggest that the >6 cm criterion lacks clinical utility. Substantial nodal enlargement remains a plausible indicator of advanced nodal burden, and a straightforward size-based rule may enhance communication and promote staging consistency. The limitation is more nuanced: existing evidence does not establish 6 cm as a definitive biological inflection point, nor does it confirm maximum diameter as the optimal surrogate for nodal tumor burden. Thus, the >6 cm criterion may be most appropriately regarded as a pragmatic historical threshold rather than a biologically definitive boundary.

In NPC, a large nodal burden may also indicate a greater likelihood of occult metastatic dissemination, particularly in EBV-associated disease. If validated, volumetric nodal assessment may better identify patients at high risk of distant failure than unidimensional diameter alone, with potential implications for treatment intensification, systemic therapy selection, and risk-adapted clinical trial design.

Future investigations of nodal size in NPC would be strengthened by initially modeling nodal diameter and nodal volume as continuous variables, ideally employing flexible statistical approaches such as restricted cubic splines, fractional polynomials, or penalized spline-based Cox models [9,10]. Should candidate thresholds be identified, they ought to be benchmarked against continuous models and alternative representations of nodal burden, using discrimination, calibration, reclassification, and clinical utility metrics—including decision-curve analysis, where appropriate [9,10,11]. Only through such a sequential and methodologically rigorous process can nodal-size thresholds be considered defensible in contemporary NPC staging.

## 6. The Caudal-Border-of-Cricoid Criterion: Reproducible Landmark Versus Biological Boundary

The caudal border of the cricoid cartilage has become a central anatomical landmark in contemporary NPC N staging. Its incorporation reflects an effort to improve the reproducibility of lower-neck nodal classification by replacing the less consistently defined supraclavicular fossa criterion used in earlier staging systems [3,4]. From an operational perspective, this modification is understandable: the cricoid cartilage is readily identifiable on cross-sectional imaging, and its caudal border provides a simple horizontal reference plane for distinguishing nodal disease extending into the lower neck. Such clarity is valuable for radiologists, radiation oncologists, multidisciplinary communication, registry documentation, and clinical trial stratification.

However, operational reproducibility should not be conflated with biological threshold validity. The caudal border of the cricoid cartilage is an anatomical landmark, not a demonstrated biological boundary of lymphatic dissemination. NPC nodal spread follows recognizable regional patterns, with frequent involvement of retropharyngeal and upper cervical nodal regions and progressive extension along cervical lymphatic pathways [17,18]. Yet there is no established lymphatic or tumor-biological mechanism indicating that nodal disease immediately below the cricoid plane represents a categorically different state from nodal disease immediately above it. Therefore, although the cricoid-border criterion may improve staging consistency, its biological status remains that of a pragmatic landmark rather than a proven risk inflection.

This distinction is particularly important because inferior nodal extent is better conceptualized as an ordinal or spatially continuous variable. Risk may plausibly increase as nodal disease extends from upper cervical levels toward lower cervical or supraclavicular regions, but this does not necessarily imply an abrupt prognostic transition at the caudal border of the cricoid cartilage. A categorical boundary may therefore simplify a continuous anatomical process. This simplification is useful for staging, but it may also introduce threshold artifacts, especially for nodes located near the boundary.

**Figure 3:**
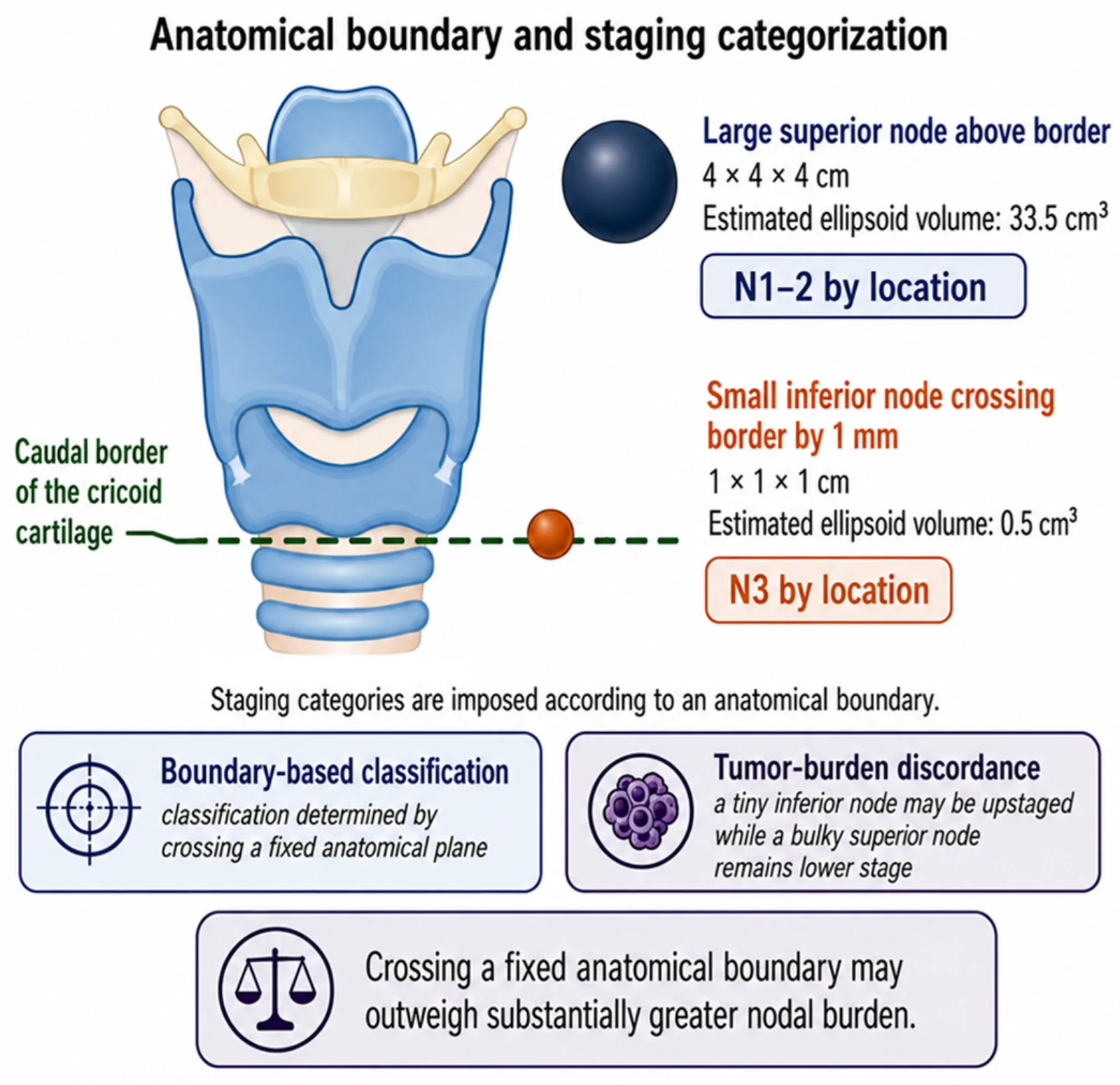
Conceptual limitation of the cricoid-border criterion in nasopharyngeal carcinoma N3 staging. Note: This schematic does not represent patient-level outcome data or a fitted statistical model. It illustrates how a fixed anatomical boundary may create discordance between nodal staging category and nodal tumor burden. A large superior node located above the caudal border of the cricoid cartilage may remain N1–2 by location, whereas a much smaller inferior node located just below the boundary may satisfy the location-based N3 criterion, assuming no other N3-defining features are present. The displayed volumes are illustrative geometric and ellipsoid estimates derived from the shown dimensions and do not represent imaging-segmented nodal volumes. This scenario highlights how crossing a fixed anatomical boundary may outweigh substantially greater nodal burden when determining the assigned N category.

The binary nature of the cricoid-border rule can generate biologically counterintuitive classifications. For example, a bulky node located immediately above the caudal border of the cricoid cartilage may be assigned a lower N category than a substantially smaller node located immediately below it, unless other N3-defining criteria are present. Such scenarios do not invalidate the criterion at the population level, but they illustrate the potential discordance between categorical anatomical staging and individual-level biological tumor burden. The central question is therefore not whether lower-neck extension is adverse, but whether the caudal border of the cricoid cartilage is the optimal boundary for defining that adverse risk. This potential discordance between boundary-based staging and nodal tumor burden is illustrated in Figure 3.

The eighth-edition proposal supported using the caudal border of the cricoid cartilage as a more reproducible imaging-based surrogate for supraclavicular fossa involvement in the IMRT era [3]. The ninth AJCC/UICC version retained inferior nodal extension below this landmark and further incorporated advanced *rENE* as an additional N3-defining feature [7]. These developments demonstrate the movement of NPC staging toward greater imaging-based precision. Nevertheless, the available evidence primarily validates inferior nodal extension as a categorical prognostic feature; it does not definitively establish that the cricoid border itself is the most biologically meaningful or statistically optimal threshold for inferior nodal spread.

A further limitation is that the cricoid-border rule has generally been evaluated as a predefined binary descriptor rather than as one candidate boundary within a continuous spatial risk model. In very large staging datasets, a binary anatomical feature may achieve statistical significance even when the absolute improvement in patient-level discrimination is modest. Therefore, evidence that nodal extension below the cricoid plane is prognostic does not necessarily establish that this plane is the optimal anatomical threshold. Flexible modeling of the lowest involved nodal position, including restricted cubic splines or related nonlinear approaches, would be required to determine whether risk changes abruptly at the cricoid border or increases more gradually along the cervical axis [9,10].

Emerging imaging-era evidence further supports the need to view inferior nodal extent as a graded risk descriptor rather than a single binary transition. Qin et al. recently evaluated MRI-defined middle-neck involvement in patients with stage N1–N2 NPC, defining this region as nodal involvement between the caudal border of the hyoid bone and the cricoid cartilage [19]. The study reported that middle-neck involvement provided additional risk stratification in patients who had not yet crossed the conventional N3-defining cricoid boundary [19]. This observation is methodologically relevant because it suggests that prognostically meaningful gradients of inferior nodal extent may exist above the cricoid plane. Therefore, the cricoid border may be a practical staging landmark, but not necessarily the first or only anatomical level at which inferior nodal spread becomes prognostically important.

A more rigorous evaluation of inferior nodal extent would require direct comparison of the cricoid-border rule with alternative representations of lower-neck disease. These could include ordinal nodal level, number of involved nodal levels, distance of the lowest involved node from a fixed cranial landmark, distance below the hyoid or cricoid plane, involvement of level IV/Vb or supraclavicular nodal regions, region-specific nodal volume, and integrated models incorporating nodal burden and extranodal extension. Such approaches would allow investigators to determine whether risk changes abruptly at a specific anatomical landmark or instead increases gradually with inferior extension and cumulative nodal disease.

The same methodological principles discussed earlier apply to this anatomical threshold. Inferior nodal extent should first be evaluated in ordinal or spatially continuous form, where possible. Flexible modeling approaches could then be used to explore whether the association between inferior extent and outcome is linear, nonlinear, or threshold-like [9,10]. If the caudal border of the cricoid cartilage is retained as a candidate cutoff, it should be benchmarked against alternative anatomical and quantitative descriptors using discrimination, calibration, reclassification, and clinical-utility analyses [9,10,11]. Such comparisons would clarify whether the current boundary is merely reproducible or genuinely optimal for prognostic classification.

The caudal border of the cricoid criterion should therefore be understood as a pragmatic imaging landmark that improves operational consistency, not as a proven biological demarcation. Its value lies in simplicity and reproducibility, both of which are essential for global staging systems. However, the available evidence does not fully resolve whether this exact boundary best captures the risk associated with inferior nodal extension. Future staging refinement should preserve the practical advantages of reproducible anatomical landmarks while formally testing whether spatially continuous or ordinal representations of inferior nodal spread provide superior prognostic performance and clinical utility.

## 7. Advanced Radiologic Extranodal Extension: A More Biological but Reproducibility-Dependent N3 Descriptor

Among recent developments in NPC nodal staging, the inclusion of advanced *rENE* constitutes an important conceptual evolution. In contrast to the >6 cm size criterion or the caudal border of the cricoid cartilage, extranodal extension does not simply describe nodal size or anatomical location. Instead, it denotes tumor extension beyond the nodal capsule into adjacent tissues, thereby providing insight into invasive tumor biology. Accordingly, advanced rENE may serve as a more biologically informative *N3* descriptor than purely anatomical or unidimensional thresholds [7,20,21].

The ninth edition of the AJCC/UICC system incorporated advanced *rENE* as an *N3*-defining feature, specifically referring to extension involving adjacent muscles, skin, or neurovascular bundles [7]. This refinement was supported by recent staging-validation studies demonstrating that advanced *rENE* independently predicts adverse outcomes across key clinical endpoints [7]. The inclusion of this feature reflects an ongoing shift in NPC nodal staging from a focus on size and anatomical extent to the incorporation of features suggestive of aggressive local invasion and potential systemic risk.

Multiple studies have demonstrated the prognostic significance of *rENE* in NPC. For example, Lu et al. assessed graded *rENE* on pretreatment MRI in node-positive NPC and observed that higher grades—particularly coalescent nodal masses with unequivocal extranodal extension and invasion into adjacent structures—were independently associated with increased risk of distant metastasis and mortality [20]. Notably, lower-grade extranodal extension did not consistently confer adverse prognosis, suggesting that both biological and clinical implications may vary according to the severity of *rENE* [20]. This observation is particularly pertinent to staging, as it underpins the TNM-9 decision to prioritize advanced *rENE* over minimal or equivocal findings.

Mao et al. further strengthened the severity-based conceptualization of *rENE* by evaluating MRI-defined advanced *rENE* in NPC across the training and validation cohorts. Their study supported the prognostic relevance of unequivocal high-grade *rENE*, particularly when associated with invasion of adjacent anatomical structures, and emphasized the need for standardized imaging definitions and reproducible reader assessment. This work is particularly relevant to staging refinement because it links biological plausibility, severity grading, validation, and reproducibility within a single radiologic descriptor [21].

Ai et al. also examined the utility of radiologic extranodal extension in NPC nodal staging, comparing several modified N3 definitions that incorporated extranodal extension patterns and cervical nodal necrosis [22]. In their internal cohort, the addition of advanced *rENE* to N3 improved 5-year disease-free survival prediction relative to the eighth-edition N classification, raising the C-index from 0.69 to 0.72 and enhancing the sensitivity of N3 for disease recurrence. This finding was replicated in an external cohort, in which the C-index increased from 0.65 to 0.72, and sensitivity for disease recurrence improved from 14.0% to 41.9% [22]. These results provide meaningful evidence supporting advanced *rENE* as a staging-relevant descriptor. Nevertheless, they do not eliminate the need to standardize its imaging definition, severity categories, reproducibility criteria, and incremental prognostic value beyond established nodal descriptors.

Nonetheless, the integration of *rENE* into staging introduces distinct methodological complexities. First, there are definitional challenges: extranodal extension encompasses a spectrum from subtle capsular irregularity or perinodal fat infiltration to overt invasion of adjacent muscles, skin, salivary glands, or neurovascular structures [20,22]. These categories may differ considerably in terms of biological severity, imaging detectability, and prognostic relevance. Consequently, a simple binary approach (“present” versus “absent”) may be inadequate, whereas advanced *rENE* could offer a more specific and clinically meaningful construct. Even so, the application of advanced *rENE* necessitates standardized imaging criteria to minimize variability across institutions, scanners, radiologists, and reporting systems.

A second challenge concerns reproducibility. Whereas nodal size can typically be measured with relative ease, the assessment of *rENE* requires nuanced interpretation of nodal margins, perinodal fat planes, invasion into adjacent tissues, and neurovascular involvement. These assessments may be affected by MRI quality, imaging protocols, slice thickness, contrast timing, interpreter experience, and institutional reporting conventions. In the absence of robust interobserver agreement and standardized reporting definitions, the apparent prognostic relevance of *rENE* may be difficult to translate into consistent global staging practice. Recent Head and Neck Cancer International Group consensus recommendations for imaging-detected extranodal extension further underscore this issue by emphasizing the need for harmonized terminology and diagnostic criteria across clinical practice and research [23]. Thus, *rENE* should be evaluated not only for prognostic value, but also for measurement reliability and reproducibility.

A third consideration pertains to incremental value. While a feature may demonstrate prognostic significance, its contribution beyond established N-stage descriptors, nodal volume, inferior nodal extent, or EBV DNA may be limited. Accordingly, the staging utility of advanced *rENE* should be evaluated in terms of its incremental effects on discrimination, calibration, reclassification, and clinical utility, rather than by statistical significance alone [9,10,11]. The favorable results reported by Ai et al., including improvements in C-index and sensitivity with external validation [22], are promising. Nonetheless, additional research is warranted to determine whether advanced *rENE* enhances patient-level risk prediction across diverse populations, treatment eras, imaging protocols, and systemic-treatment strategies.

An additional consideration involves the relationship between extranodal extension and nodal burden. While advanced *rENE* may, in part, reflect aggressive tumor biology, it may also be associated with larger nodal size, matted nodes, necrosis, or increased nodal volume, leading to the potential for overlapping prognostic information. For the purposes of staging refinement, the critical question is whether advanced *rENE* offers independent prognostic value beyond three-dimensional nodal burden and inferior nodal extent. Investigations that concurrently model nodal volume, nodal-level distribution, size, necrosis, inferior extent, and extranodal extension would be instrumental in determining whether advanced *rENE* is an independent biological descriptor or primarily a correlate of advanced nodal burden.

In light of these considerations, advanced *rENE* may be regarded as one of the more promising refinements in TNM-9, yet it cannot be considered methodologically complete. Its biological plausibility is stronger than that of purely geometric thresholds, as it reflects invasive behavior rather than simply exceeding a size or anatomical boundary. Nonetheless, biological plausibility alone is insufficient for widespread adoption in staging. The descriptor must also be reproducible, externally validated, incrementally informative, and clinically actionable. Consequently, future research should emphasize standardized MRI definitions, structured radiology reporting, interobserver reliability studies, and integrative models that evaluate advanced *rENE* in conjunction with nodal volume, inferior extent, EBV DNA, and host-related biomarkers.

Importantly, radiologic and pathologic extranodal extension are related but not identical entities. On MRI, irregular nodal margins may reflect inflammation, fibrosis, nodal matting, or imaging artifacts rather than definite extracapsular spread. Therefore, imaging-defined *rENE* should be viewed as an imaging correlate of invasive tumor behavior, not as a direct substitute for histopathologic extranodal extension.

In summary, advanced *rENE* constitutes a meaningful advancement toward biologically informed NPC nodal staging. By capturing invasive tumor behavior, it addresses some of the limitations inherent in size- and location-based criteria, with emerging evidence supporting its prognostic and staging relevance [7,20,21]. Nevertheless, its ultimate clinical value will depend on reproducible application and demonstrable incremental risk stratification beyond current anatomical and volumetric descriptors. Thus, advanced rENE may be considered a promising, yet validation-dependent, N3 feature.

## 8. Discussion

The principal finding of this review is that the evidence supporting the prognostic domains represented by the current N3 criteria is stronger than the evidence validating some of the specific quantitative or anatomical boundaries used to define them. Nodal size, three-dimensional nodal burden, inferior nodal extent, spatial distribution, necrosis, and advanced rENE each represent distinct aspects of nodal disease and may carry prognostic information [5,6,13,15,16,19,20,21,22]. However, demonstrating that a descriptor is prognostically relevant does not establish that a particular cutoff or anatomical boundary constitutes an optimal, reproducible, or biologically discrete risk threshold [8,9,10,11,12,24]. This distinction is particularly important for the historically inherited >6 cm nodal-size threshold and the caudal border of the cricoid cartilage, whereas advanced rENE represents a more recently developed criterion supported by contemporary imaging-based prognostic and validation studies [3,7,20,21,22,23]. The current and emerging N3-related descriptors, the risk dimensions they represent, their main limitations, and key priorities for future validation are summarized in Table 1.

A key implication of this distinction concerns the evidentiary standard required for established staging boundaries. The lack of a validated superior alternative does not, in itself, validate the existing threshold. A 6 cm cutoff is not validated simply because a 4 cm cutoff, nodal volume, or another representation has not yet been shown to be superior; likewise, the caudal border of the cricoid cartilage does not become a biological threshold solely because no alternative anatomical boundary has been validated as superior. Instead, the central question is whether the chosen boundary has been demonstrated to provide a reproducible and clinically meaningful representation of the underlying risk relationship. This principle does not suggest that established criteria lack clinical utility. Rather, it separates three related but distinct evidentiary claims: the prognostic validity of the underlying variable, the validity of the threshold used for categorization, and the pragmatic utility of that categorization within an international staging system [8,9,10,11,12,24].

The >6 cm criterion exemplifies this issue with particular clarity. While large nodal size is biologically plausible as an indicator of increased tumor burden, and extensive nodal disease has long been associated with adverse prognosis, this evidence supports the relevance of nodal burden rather than the specific validity of the 6 cm cutoff. The 6 cm threshold predates the widespread use of MRI-based nodal assessment and has persisted across multiple staging editions as a clinically recognizable marker of bulky cervical adenopathy [4]. Although this historical continuity offers advantages for clinical communication and cross-era comparison, continued inclusion in staging systems does not equate to evidence that this value represents a biological or statistical inflection point. A threshold may retain clinical utility without necessarily representing a biologically discrete boundary, but these characteristics must not be conflated.

Contemporary MRI-era studies further underscore that the prognostic significance of nodal size should not be conflated with validation of a specific diameter threshold. Huang et al. identified MRI-derived maximal axial nodal diameter, proposing 4 cm as a potentially more informative prognostic cutoff, whereas Ni et al. reported that maximum cervical nodal diameter was not independently prognostic in locoregionally advanced NPC treated with IMRT [5,13]. These seemingly divergent findings may stem from differences in cohort characteristics, endpoint definitions, disease stage distribution, measurement techniques, covariate adjustment, and methods of cutoff determination. Crucially, these studies do not establish 4 cm as an appropriate substitute for 6 cm. Instead, they highlight that the association between unidimensional nodal size and clinical outcomes remains insufficiently characterized to establish the historically inherited 6 cm boundary—or any proposed alternative diameter cutoff—as an optimally validated categorical threshold.

Evidence from volumetric studies further reinforces this interpretation. Yuan et al. demonstrated the prognostic significance of cervical nodal volume, Chen et al. identified cervical nodal tumor volume as prognostically relevant in patients with nodal metastases, and Liang et al. demonstrated the prognostic significance of total tumor volume in IMRT-treated NPC [6,15,16]. These studies varied in segmentation techniques, anatomical components included in volumetric assessment, imaging platforms, clinical endpoints, and proposed cutoff values; thus, they do not provide sufficient justification to replace the 6 cm threshold with a single volumetric cutoff. The collective value of these investigations lies in their consistent demonstration that three-dimensional tumor burden carries prognostic information that may not be fully captured by a single maximum diameter. Therefore, current evidence supports nodal burden as a prognostic domain more strongly than it substantiates any specific diameter- or volume-based cutoff as a uniquely valid categorical threshold.

The conceptual implications of this discordance are significant. Reducing a three-dimensional, often irregular nodal process to a single maximum dimension oversimplifies the underlying anatomical and volumetric complexity of nodal disease. Lymph nodes with similar maximal diameters can differ markedly in volume, morphology, necrosis, matting, and anatomical distribution, while nodes immediately above and below a numerical cutoff may have nearly indistinguishable underlying disease burdens. Dichotomizing nodal size therefore imposes an artificial categorical threshold on a variable whose association with risk may actually be continuous or nonlinear [8,9,12,24]. This limitation does not preclude categorization for staging purposes, as international staging systems must necessarily distill biological complexity into practical criteria. Nevertheless, selection of a simplified boundary should be justified by its demonstrated prognostic performance, reproducibility, and clinical utility, rather than by the presumption that it reflects a natural biological breakpoint.

Additional MRI-defined nodal characteristics further underscore the multidimensional complexity of nodal risk. Liu et al. showed that nodal grouping—defined as three or more contiguous lymph nodes within a single nodal region—was independently associated with multiple survival outcomes and may provide additional prognostic granularity for N classification and treatment stratification [25]. Cervical nodal necrosis represents another pertinent phenotype; a systematic review and meta-analysis by Ai et al. demonstrated its adverse prognostic association in nasopharyngeal carcinoma treated with intensity-modulated radiotherapy [26]. At present, neither nodal grouping nor nodal necrosis should be considered a validated replacement for existing N3 criteria. Rather, their main relevance to this discussion is conceptual: patients with identical largest nodal diameters and similar relationships to the cricoid plane may nonetheless differ markedly in aggregate nodal burden, number and distribution of involved nodes, extent of necrosis, degree of matting, and invasive phenotype. These findings further illustrate the limitations of relying on any single unidimensional or positional descriptor to fully capture the risk profile of advanced nodal disease.

A parallel methodological issue arises with the definition of inferior nodal extent. Replacing the supraclavicular fossa criterion with extension below the caudal border of the cricoid cartilage improved anatomical clarity and enhanced imaging-based reproducibility [3]. This shift represented an important advance, as a staging criterion that cannot be applied consistently across observers and institutions offers limited value for international classification. However, reproducibility of an anatomical landmark and the biological validity of the boundary defined by that landmark are distinct considerations. While the caudal border of the cricoid cartilage serves as a readily identifiable horizontal reference plane, there is no established biological rationale demonstrating that nodal extension immediately below this plane represents a categorically different disease state compared with otherwise similar nodal involvement immediately above it.

The recent study by Qin et al. is especially informative in this context. MRI-defined middle-neck involvement—situated between the caudal borders of the hyoid and cricoid cartilages—provided additional prognostic stratification among patients classified as N1–N2, who therefore did not meet the conventional location-based N3 criterion [19]. This finding neither invalidates the cricoid border nor establishes the hyoid or middle-neck region as a superior alternative. Instead, it demonstrates that prognostically meaningful information regarding inferior nodal dissemination exists above the current binary threshold. The emerging biological interpretation aligns more closely with the concept of a spatial risk gradient than with the assumption that adverse risk begins only when a node crosses a single predetermined horizontal plane.

This distinction carries important practical implications for interpreting the cricoid criterion. Its principal strength may be operational rather than biological: it is more easily and reliably identified on cross-sectional imaging than the former supraclavicular fossa designation, thereby enhancing staging reproducibility [3]. While operational reproducibility provides a legitimate justification for retaining an anatomical staging landmark, if this is the principal rationale, the cricoid criterion should be viewed as a pragmatic marker of inferior nodal dissemination rather than as a validated biological threshold. Accordingly, future analyses should assess inferior nodal extent using ordinal or spatially continuous frameworks—including nodal level, number of involved levels, distance-based inferior extent, or region-specific nodal burden—before determining whether the cricoid plane is indeed the most informative boundary for N-stage categorization [9,10,19].

Advanced rENE occupies a distinctly stronger evidentiary position. In contrast to nodal diameter or crossing a fixed anatomical boundary, rENE represents overt tumor invasion beyond the nodal capsule and therefore has a more direct biological association with aggressive tumor behavior. Lu et al. demonstrated that the prognostic impact of MRI-defined rENE was severity dependent, with advanced patterns showing stronger associations with distant metastasis and mortality than more subtle abnormalities [20]. Mao et al. subsequently demonstrated that unequivocal advanced MRI-defined rENE was associated with worse outcomes across both training and validation cohorts, while underscoring the importance of standardized radiologic assessment [21]. Ai et al. further reported that incorporating advanced rENE into modified N3 definitions improved disease-free survival prediction and increased sensitivity for recurrence in both internal and external cohorts [22]. Collectively, this body of evidence supports the incorporation of advanced rENE into the ninth edition of the AJCC/UICC N classification [7].

The experience with advanced rENE is methodologically instructive, exemplifying a more robust progression from candidate descriptor to incorporated staging criterion. Biological plausibility for rENE was accompanied by severity characterization, prognostic evaluation, multi-cohort validation, and assessment of incremental staging performance [20,21,22]. However, rENE also illustrates that biological relevance alone does not suffice. Interpretation remains influenced by MRI acquisition protocols, diagnostic definitions, and interobserver variability, prompting international consensus recommendations for standardized terminology and imaging criteria [23]. As a result, advanced rENE presently offers a comparatively stronger evidentiary foundation than either the historically inherited 6 cm threshold or the primarily anatomical cricoid-border criterion, yet it still requires ongoing efforts to ensure measurement reproducibility and international implementation.

Taken together, these findings demonstrate that current N3-defining criteria should not be presumed to have equivalent evidentiary maturity simply because they share the same staging designation. The >6 cm criterion remains primarily a historically derived surrogate for bulky nodal burden; the cricoid-border criterion offers a reproducible anatomical marker of inferior nodal dissemination; and advanced rENE represents invasive nodal behavior substantiated by contemporary prognostic evaluation and validation. Recognizing this hierarchy does not necessitate the immediate removal of the first two criteria. Instead, it clarifies the distinction between staging utility and threshold validation, highlighting where the evidentiary foundation is strongest and where criterion-specific reappraisal is most needed.

**Figure 4:**
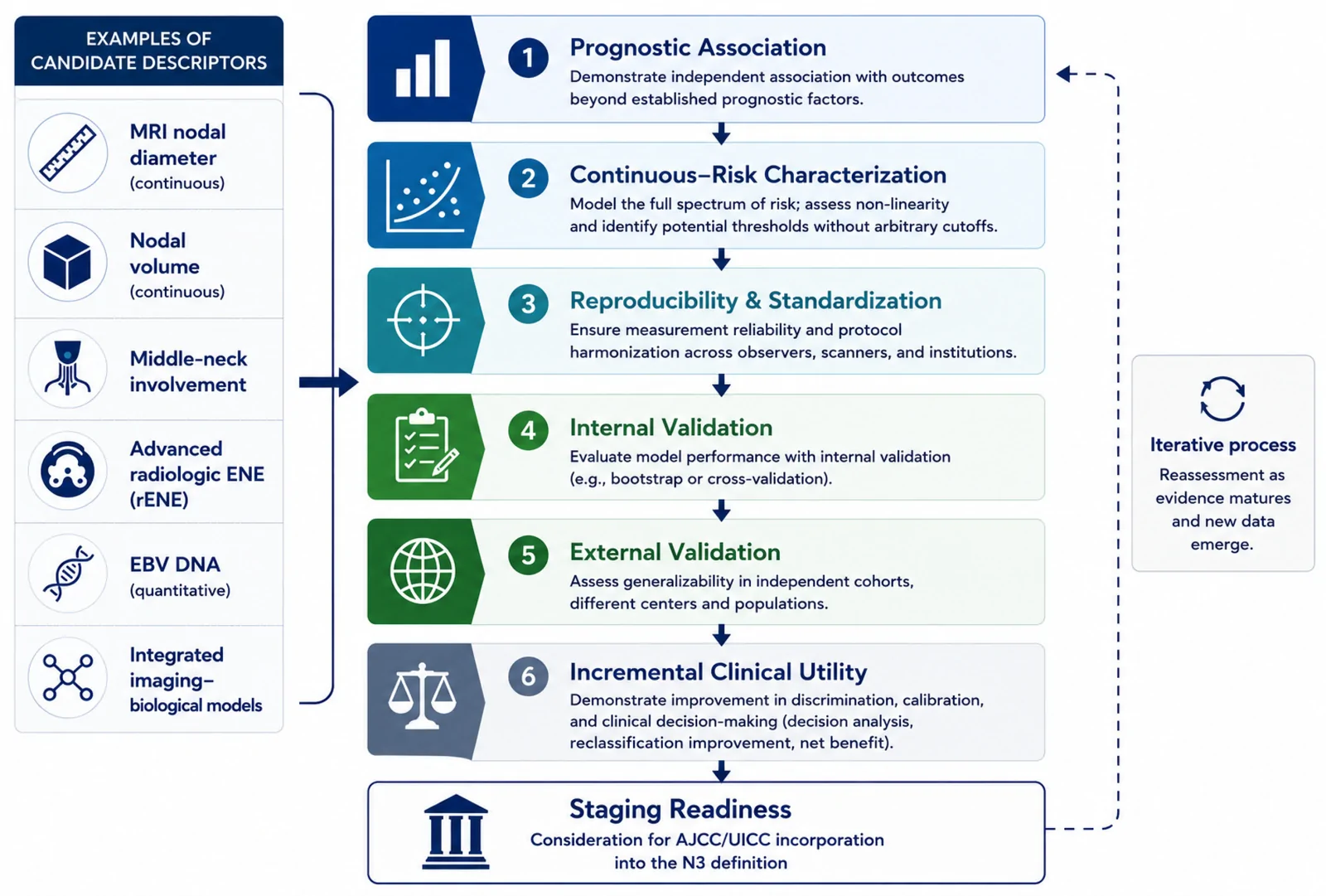
Hierarchy of evidence for evaluating candidate descriptors for future refinement of the AJCC/UICC nasopharyngeal carcinoma N3 classification. Candidate descriptors proposed for refinement of the AJCC/UICC N3 classification should progress through a sequential evidential pathway before consideration for incorporation into a staging system. Independent prognostic association represents the initial requirement but is insufficient in isolation. Subsequent evaluation should include characterization of the continuous risk relationship, assessment of measurement reproducibility and standardization, internal and external validation, and demonstration of incremental clinical utility beyond the current staging system. Candidate descriptors that successfully satisfy these evidential requirements may be considered for future AJCC/UICC staging refinement. Because scientific evidence continues to evolve, this process should be regarded as iterative rather than linear. Note: Demonstration of prognostic significance alone is necessary but not sufficient for incorporation into a cancer staging system. Abbreviations: AJCC, American Joint Committee on Cancer; EBV, Epstein–Barr virus; ENE, extranodal extension; MRI, magnetic resonance imaging; rENE, radiologic extranodal extension; UICC, Union for International Cancer Control.

The implications extend beyond the individual N3 descriptors discussed here. Incorporating a candidate variable into an international staging system requires a substantially higher evidentiary standard than simply demonstrating statistical significance in a prognostic factor study. A descriptor may be independently prognostic and clinically informative without being appropriate for inclusion in TNM staging. A staging-ready criterion should demonstrate a robust, well-characterized association with clinical outcomes, reproducible measurement, internal and external validity, incremental prognostic value beyond existing categories, clinical utility, and sufficient feasibility to justify routine categorical use [8,9,10,11,12]. For continuous or ordinal variables, the nature of the underlying risk relationship should also be carefully characterized before imposing a categorical boundary. The proposed hierarchy of evidence necessary for progression from candidate prognostic descriptor to staging-ready criterion is summarized in Figure 4.

Importantly, these evidentiary requirements must be applied symmetrically. Currently, none of the proposed imaging-derived, volumetric, spatial, or integrated alternatives is supported by sufficient evidence to replace the established N3 criteria with a specific new threshold or anatomical boundary. However, the inability of a proposed alternative to meet the standards for staging incorporation does not constitute affirmative validation of the existing criterion. It would be methodologically inconsistent to require rigorous continuous-risk characterization, external validation, reproducibility, and incremental clinical utility from every proposed new descriptor while treating an established boundary as validated solely on the basis of historical precedent. While historical criteria deserve acknowledgment for their clinical experience, reproducibility, continuity, and global familiarity, these attributes cannot substitute for direct evidence supporting the validity of the criterion itself.

This principle is especially evident when considering negative evidence related to proposed alternatives. If a 4 cm threshold fails external validation, this finding argues against 4 cm; it does not validate 6 cm. If a volumetric cutoff demonstrates inconsistent performance across cohorts, this challenges that specific volumetric categorization but does not establish maximum diameter as the optimal representation of nodal burden. Likewise, the inability to identify an anatomical landmark superior to the cricoid plane does not imply that biological risk changes precisely at the cricoid border. The evidentiary status of each candidate—including established criteria—must therefore be judged based on the strength of evidence supporting that candidate itself. This principle is particularly important in cutoff research, where data-driven optimization can yield seemingly convincing thresholds that are unstable or perform poorly when applied outside the derivation cohort [8,9,10,11,12,24].

At the same time, validating a staging boundary should not be conflated with the need to identify a single biological discontinuity. Cancer risk typically varies along a continuum, while staging systems must necessarily impose categorical boundaries. A threshold can remain clinically justifiable even in the absence of an abrupt biological transition, provided its selection yields a reproducible and clinically meaningful simplification of the underlying risk distribution. Ideally, such justification should rest on comparative prognostic performance, calibration, clinical utility, stability across patient populations, and feasibility of implementation—not merely statistical significance [9,10,11]. In the absence of a demonstrated biological inflection point, the proper interpretation is not that categorization should be abandoned, but rather that the resulting boundary should be recognized as a pragmatic staging construct, not a biologically definitive division.

Emerging imaging and biological models are especially informative from this perspective. Zhou et al. demonstrated that anatomical tumor spread, tumor volume, and plasma EBV DNA each contributed complementary prognostic information in NPC [27]. Similarly, Chen et al. showed that integrating tumor volume with pretreatment plasma EBV DNA further refined prognostic stratification beyond anatomical descriptors alone [28]. These findings do not justify replacing conventional N staging with integrated imaging–biological models. Rather, they highlight that anatomical extent, three-dimensional tumor burden, and biological tumor load represent partially complementary risk domains. Such models provide valuable benchmarks for assessing the prognostic information retained—and the information lost—by simplified categorical N-stage definitions.

Within this context, quantitative plasma EBV DNA should be distinguished from tissue-based EBV status. Pretreatment plasma EBV DNA serves as a circulating biomarker reflecting viral tumor burden and has demonstrated prognostic significance in NPC, whereas tissue-based EBV assessment primarily establishes the EBV-associated status of the malignancy [1,29,30]. This distinction is important when integrating biological data with anatomical N-stage descriptors, given that anatomical nodal burden and circulating viral tumor burden capture different, albeit related, dimensions of disease risk.

A multidimensional perspective has become increasingly relevant in contemporary NPC, as modern imaging enables detailed characterization of nodal disease beyond maximum diameter alone. Variables such as total nodal volume, number and grouping of involved nodes, necrosis, laterality, inferior extent, and extranodal extension each define distinct facets of the regional disease process [5,6,15,16,19,20,21,22,25,26]. Currently, there is no evidence to support incorporation of all these variables into TNM staging, and doing so risks undermining the simplicity and global applicability that are key strengths of the staging system. The central aim should not be maximal prognostic complexity, but rather the identification of descriptors that are sufficiently reproducible, nonredundant, and clinically meaningful to warrant inclusion in a streamlined staging framework.

Implementation requirements are therefore as critical as statistical performance. Volumetric segmentation requires additional imaging and segmentation infrastructure together with standardized contouring protocols; plasma EBV DNA assessment requires assay standardization; and evaluation of rENE depends on imaging quality, interpretation criteria, and radiologic expertise [23,30]. These resources are not universally available across institutions or health systems. Thus, a descriptor that demonstrates strong prognostic performance in specialized datasets but cannot be measured reproducibly or implemented broadly may be better suited to individualized prognostic modeling than to inclusion in an international TNM classification. Global applicability must remain a primary consideration when increasingly sophisticated imaging and biological markers are proposed for staging refinement.

For these reasons, future N3 refinement should not be limited to substituting 6 cm with 4 cm, the cricoid plane with another horizontal landmark, or a diameter threshold with a single volumetric cutoff. Such substitutions risk perpetuating the same methodological limitations in a different guise. Candidate descriptors should first be evaluated in their native continuous, ordinal, volumetric, or severity-graded forms, employing flexible statistical methods where appropriate to characterize the underlying risk relationships [8,9,10,12,24]. If categorization is deemed necessary for clinical application, the proposed boundary should be rigorously assessed for stability, external validity, incremental prognostic performance, reproducibility, and clinical utility [9,10,11]. External validation should extend beyond replication in a secondary dataset to include evaluation of transportability across diverse populations, imaging protocols, treatment eras, systemic-therapy approaches, and outcome distributions [9,10]. This approach ensures that categorization is guided by evidence rather than allowing preselected categories to dictate the analytic framework.

The clinical significance of this distinction is substantial. N classification is not merely a descriptive label; it underpins stage grouping, prognostic communication, clinical-trial stratification, outcome reporting, and the identification of patient subgroups for potential treatment intensification [2,3,7]. An imposed categorical boundary can influence how patients with biologically similar disease are represented in clinical datasets and how outcomes are interpreted across N categories. Conversely, excessive staging complexity that yields only marginal prognostic improvement may undermine reproducibility and global applicability. Future refinement must therefore balance two critical objectives: maintaining the simplicity necessary for an international staging system and ensuring that its categorical boundaries remain justifiable in light of contemporary anatomical, biological, and statistical evidence.

Although this review centers on N-stage classification at initial diagnosis, baseline TNM stage also carries important implications for subsequent recurrence risk. Patients with more advanced nodal disease generally experience a higher risk of disease recurrence, particularly distant metastasis. However, recurrent NPC is shaped by multiple factors beyond initial TNM stage and thus necessitates independent clinical evaluation, risk stratification, and management.

In summary, the existing literature does not support a binary choice between uncritical retention of current N3 criteria and wholesale adoption of proposed alternatives. Instead, it supports a more nuanced conclusion. While the prognostic significance of nodal burden, inferior nodal dissemination, and invasive extranodal behavior is well established, the evidence validating the specific categorical representations of these domains remains heterogeneous. The 6 cm criterion serves as a pragmatic historical marker of bulky nodal disease; the cricoid border offers a reproducible operational reference for inferior extension; and advanced rENE exemplifies the most fully developed contemporary pathway from biological rationale to prognostic validation and staging integration. Future progress should therefore prioritize not simply the identification of new cutoffs, but the rigorous evaluation of whether existing and proposed boundaries accurately and reproducibly translate the continuous and multidimensional character of nodal risk into clinically useful staging categories. This methodological perspective underpins the subsequent consideration of anatomical N staging within the broader biological context of NPC.

## 9. Biological Context Beyond Anatomical N Staging

Anatomical N staging delineates the distribution and extent of nodal disease, yet it does not fully capture the biological context of nodal metastasis. This limitation is especially pertinent in NPC, where tumor behavior is closely associated with EBV biology and systemic tumor burden [1,29,30]. Therefore, refinement of N3 criteria should be regarded as a single facet of risk stratification, rather than a comprehensive approach to individualized prognostication.

Plasma EBV DNA is the most extensively validated biomarker in NPC, providing prognostic information beyond conventional anatomical staging. Leung et al. demonstrated that pre-therapy circulating EBV DNA provides complementary prognostic value to TNM staging in NPC [29]. Subsequent systematic reviews and consensus recommendations have reinforced the clinical significance of plasma EBV DNA while also highlighting ongoing challenges in assay standardization, interlaboratory reproducibility, and global implementation [30]. These considerations are germane to N-stage refinement, as patients with comparable anatomical N categories may exhibit substantial heterogeneity in systemic viral tumor burden. Likewise, nodal burden in NPC may reflect not only the extent of regional disease but also a greater propensity for occult metastatic dissemination, such that bulky nodal disease may serve as an anatomical surrogate for higher systemic tumor burden.

This distinction underscores the central premise of this review: anatomical categories, including N3 thresholds, should not be interpreted as comprehensive biological risk models. Patient outcomes may be influenced not only by nodal size, inferior extent, volume, or extranodal extension, but also by systemic tumor burden and host–tumor biology. Additional host-related factors—such as inflammatory, nutritional, or oxygenation parameters—may also be prognostically relevant; however, their incorporation into staging frameworks would require rigorous standardization, validation, and evidence of incremental clinical utility [9,10,11].

Accordingly, the aim of refining N3 criteria should not be to convert the TNM staging system into an exhaustive biomarker-based prediction model. Rather, the objective is to ensure that anatomical thresholds are both biologically and statistically defensible, while recognizing that individualized risk assessment in NPC may require integration of anatomical, imaging-derived, viral, and host-related parameters. This perspective maintains the intended scope of the review, while acknowledging that nodal staging constitutes only one element within a broader, multifaceted prognostic framework.

## 10. Proposed Methodological Framework for Future N3 Refinement

Future refinement of NPC N3 staging would benefit from explicitly distinguishing the clinical utility of existing categories from the threshold validity of the boundaries that define them. The current criteria—including the >6 cm nodal-size threshold, extension below the caudal border of the cricoid cartilage, and advanced *rENE*—offer a practical framework for staging and communication. Nevertheless, further refinement requires careful evaluation of whether these descriptors represent optimal, reproducible, and clinically meaningful risk boundaries, rather than categories primarily supported by statistical associations, operational convenience, or historical continuity. A proposed stepwise methodological framework for evaluating candidate N3 refinements is summarized in Table 2.

A rigorous methodological framework should initially analyze candidate nodal descriptors in their native form. Nodal diameter and nodal volume are best evaluated as continuous variables; inferior nodal extent is more appropriately treated as an ordinal or spatially continuous descriptor; and *rENE* may be better assessed with standardized severity grading rather than binary definitions. Flexible modeling strategies—such as restricted cubic splines, fractional polynomials, or penalized spline-based Cox regression—can elucidate whether risk relationships are linear, nonlinear, gradual, or characterized by reproducible inflection points [9,10]. This analytic step is essential prior to adopting simplified categorical thresholds.

Second, candidate thresholds ought to be derived only after the underlying continuous or ordinal risk relationship has been adequately characterized. Where a genuine inflection point is identified, threshold-based simplification may be clinically warranted. Conversely, if risk demonstrates a smooth, continuous increase, categorical boundaries should be recognized as pragmatic constructs rather than as biologically discrete divisions. Accordingly, any proposed cutoffs—whether based on nodal diameter, volume, anatomical level, distance from an imaging landmark, or advanced extranodal extension—should be interpreted in relation to the empirical risk curve, rather than selected solely on the basis of statistically significant group separation [8,9,12].

Third, threshold selection should incorporate safeguards to mitigate overfitting. Data-driven cutoffs are susceptible to optimism bias, particularly when multiple candidate boundaries are examined. Internal validation methods, such as bootstrapping or cross-validation, are necessary to estimate optimism-corrected performance [9,10]. Nevertheless, internal validation alone is insufficient for broad adoption of staging. Candidate thresholds must also undergo external validation in cohorts that differ in geography, endemicity, imaging protocols, EBV DNA availability, treatment era, systemic therapy approaches, and follow-up practices. This is particularly salient in NPC, where endemic and non-endemic populations may exhibit differences in EBV association, clinical presentation, treatment access, and outcome distributions.

Fourth, validation should not be predicated solely on *p*-values. In large staging datasets, minor differences may achieve statistical significance without imparting clinically meaningful enhancements in individual-level risk stratification. Thus, candidate N3 refinements should be appraised using absolute improvements in discrimination, calibration, reclassification, and clinical utility. Relevant metrics might include Harrell’s C-index, time-dependent area under the curve, likelihood-based measures, Brier score, calibration plots, net reclassification indices, and decision-curve analysis where appropriate [9,10,11]. Crucially, the magnitude of improvement should be evaluated from a clinical, not merely statistical, perspective.

Fifth, proposed categorical refinements should be systematically compared with continuous and integrated alternatives. For instance, a 6 cm diameter threshold should be benchmarked against continuous nodal diameter, MRI-based maximal axial diameter, nodal volume, total nodal burden, and composite size-volume models. Similarly, the caudal border of the cricoid criterion should be evaluated against ordinal nodal level, distance-based inferior extent, the number of involved nodal levels, and region-specific nodal volume. Advanced *rENE* should be assessed alongside nodal volume, necrosis, nodal matting, inferior extent, and EBV DNA to determine whether it provides independent and incremental prognostic value beyond existing descriptors [5,6,15,16,19,20,21,22,23].

Sixth, reproducibility must be treated as a core component of staging validity. A descriptor that improves prognostic performance in expert-center datasets may not be suitable for global staging if it cannot be measured consistently. Nodal diameter is relatively simple to measure, whereas nodal volume requires segmentation, and *rENE* requires interpretive judgment. Future studies should therefore report interobserver agreement, imaging protocol requirements, segmentation methods, and structured reporting criteria. For advanced *rENE*, reproducibility is particularly important because subtle extranodal irregularity, nodal matting, frank invasion of adjacent structures, and neurovascular involvement may not be interpreted uniformly across readers or institutions [20,22].

Seventh, clinical actionability should be prioritized before incorporating new staging descriptors. An N3 refinement ought to contribute not only to improved model performance but also to enhanced clinical decision-making—informing prognosis, trial stratification, systemic treatment intensification, surveillance strategies, or patient counseling. Decision-curve analysis and related clinical utility assessments can elucidate whether a new descriptor substantially improves decision-making across relevant risk thresholds [11]. A statistically favorable, yet clinically impractical, descriptor may be more appropriate for individualized prognostic models than for formal inclusion in TNM staging.

Eighth, future staging models should strive to maintain the TNM system’s core strengths while addressing its recognized limitations. The enduring utility of TNM lies in its simplicity, global applicability, and historical continuity. Therefore, forthcoming N3 refinements should avoid unnecessary complexity and supplanting staging with opaque predictive models. A pragmatic approach entails retaining an anatomically based staging foundation, while periodically benchmarking it against imaging-derived and biologically informed alternatives. Only descriptors that demonstrate reproducibility, external validation, and clinically meaningful incremental value should be considered for integration into future staging systems.

**Figure 5:**
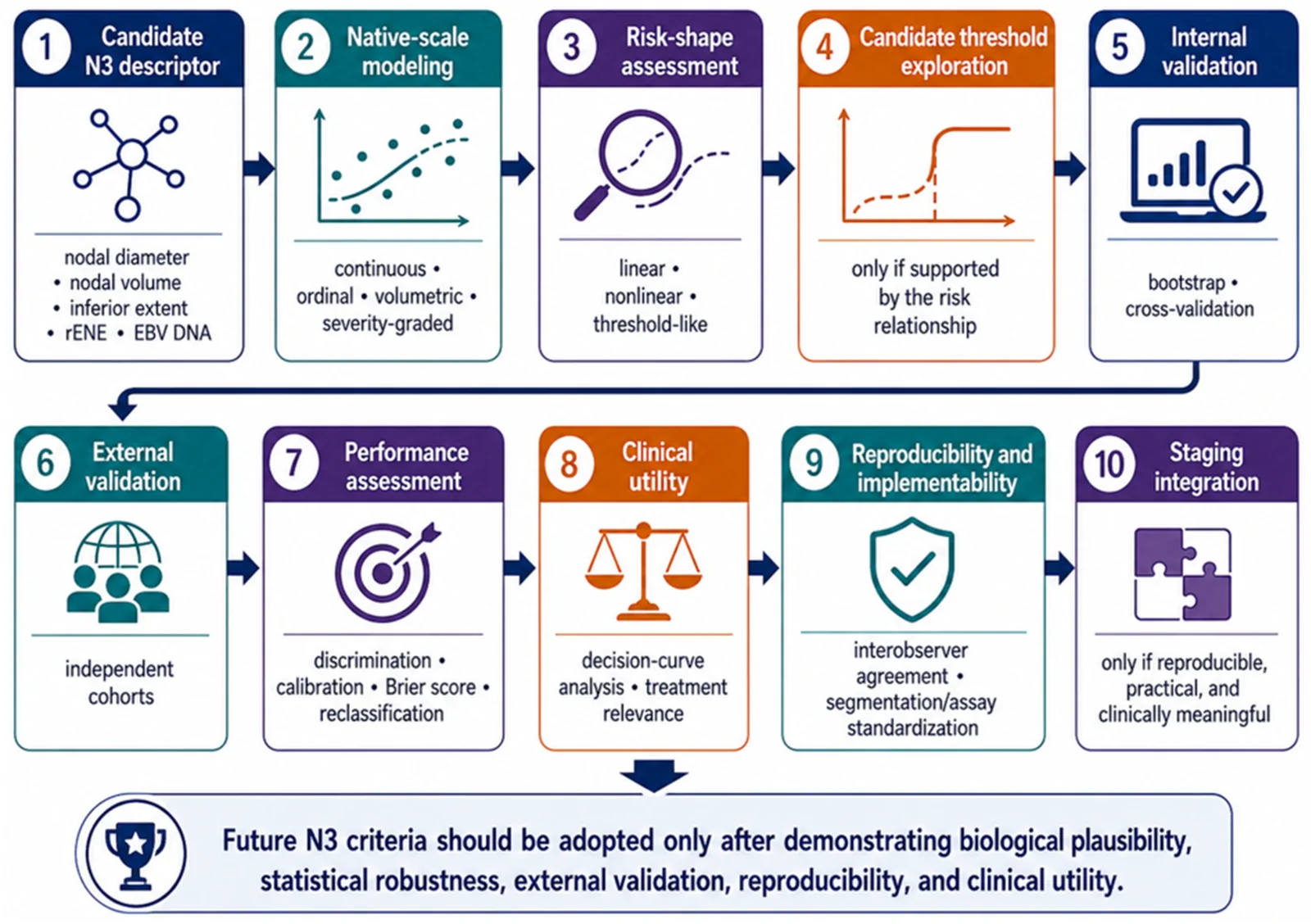
Proposed methodological roadmap for future refinement of N3 criteria in nasopharyngeal carcinoma. Note: Candidate N3 descriptors should undergo a stepwise validation pathway before incorporation into staging. The process begins with an explicit definition of the candidate descriptor, followed by native-scale modeling, assessment of the underlying risk relationship, and exploration of candidate thresholds only when supported by the empirical risk pattern. Subsequent steps should include internal validation, external validation, comprehensive performance assessment, clinical-utility evaluation, reproducibility and implementability testing, and final consideration for staging integration. Candidate criteria should be adopted only if they demonstrate biological plausibility, statistical robustness, external validity, reproducibility, clinical utility, and practical feasibility.

This proposed framework reconceptualizes N3 refinement as a stepwise validation challenge, rather than a search for a single optimal cutoff. The principal question is not simply whether a 6 cm threshold should be replaced by 4 cm, the cricoid plane by another anatomical landmark, or nodal diameter by volume. Instead, the essential inquiry is whether any proposed descriptor or threshold more accurately characterizes the continuous, multidimensional, and biologically heterogeneous nature of nodal risk in NPC. Accordingly, future staging research should move beyond categorical association testing and focus on continuous-risk characterization, interobserver reproducibility, external validation, and demonstration of clinical utility. This proposed validation pathway is presented schematically in Figure 5.

## 11. Limitations of This Review

Several limitations should be acknowledged in the context of this review. First, this work constitutes a narrative and methodological review rather than a systematic review or meta-analysis. As such, while the discussion is informed by key literature on staging, imaging, and methodology, it does not provide pooled estimates or formally grade the certainty of the evidence. The primary objective is not to quantify the prognostic impact of individual N3-related descriptors, but rather to critically assess whether the thresholds currently used to define advanced nodal disease align with contemporary standards for threshold validation.

Second, the critique advanced herein should not be construed as evidence that the current AJCC/UICC N3 criteria lack clinical utility. The >6 cm nodal-size threshold, extension below the caudal border of the cricoid cartilage, and advanced *rENE* each address clinically relevant dimensions of nodal risk. The central concern is narrower: existing evidence does not conclusively establish that the precise thresholds employed for these criteria correspond to biological inflection points or represent statistically optimal cutoffs.

Third, the alternative descriptors considered in this review—including MRI-based maximal axial diameter, nodal volume, middle-neck involvement, total tumor volume, and advanced rENE—ought not to be regarded as definitive replacements for current staging criteria. Each candidate descriptor necessitates further validation, standardization, reproducibility assessment, and demonstration of incremental clinical utility before its integration into formal staging frameworks can be substantiated.

Fourth, the available literature exhibits considerable heterogeneity with respect to patient populations, endemic versus non-endemic settings, imaging protocols, radiotherapy techniques, systemic therapy strategies, EBV DNA availability, duration of follow-up, and statistical approaches. Such variability may affect the observed performance of nodal descriptors and constrain the generalizability of specific thresholds across diverse institutions and populations.

Finally, several proposed methodological approaches—including continuous spatial modeling of inferior nodal extent, integrated imaging–biological risk models, and flexible nonlinear modeling of nodal burden—remain, at present, more conceptual than validated as practical staging tools. Their eventual adoption within NPC staging will depend on their demonstrated capacity to enhance reproducibility, calibration, clinical utility, and global implementability. At the same time, any refinement must preserve the simplicity that underpins the clinical utility of TNM staging systems.

## 12. Conclusions

The current AJCC/UICC N3 criteria for NPC offer a practical, reproducible, and globally applicable framework for identifying patients with advanced nodal disease. The >6 cm nodal-size threshold, extension below the caudal border of the cricoid cartilage, and advanced *rENE* each address clinically relevant aspects of nodal risk. Nonetheless, the evidential basis for these descriptors is not uniform, and their observed prognostic associations should not be construed as definitive evidence that the specific thresholds defining N3 disease represent biological inflection points or statistically optimal boundaries.

The >6 cm criterion illustrates the limitations inherent in the inheritance of historical thresholds. While substantial nodal enlargement remains clinically plausible as an indicator of advanced tumor burden, the specific 6 cm threshold was established prior to the advent of contemporary MRI-based staging and may insufficiently capture the three-dimensional complexity of nodal disease. Similarly, defining inferior nodal extension by the caudal border of the cricoid cartilage enhances anatomical reproducibility relative to earlier lower-neck definitions, yet it remains a pragmatic imaging landmark rather than a validated biological boundary of lymphatic dissemination. In contrast, advanced *rENE* offers a more biologically informative perspective by reflecting invasive tumor behavior; however, its value in staging depends on the adoption of standardized imaging definitions, interobserver agreement, external validation, and demonstrated incremental clinical utility.

A principal message of this review is that prognostic association does not equate to threshold validity. Analyses involving large cohorts may confirm that a categorical descriptor is associated with clinical outcomes, yet such findings do not inherently establish that the chosen cutoff or anatomical boundary is optimal. This distinction is particularly important in large datasets, where statistically significant associations may not yield clinically meaningful improvements in discrimination, calibration, reclassification, or decision utility. Accordingly, future staging research should move beyond categorical association testing and characterize nodal descriptors in continuous, ordinal, volumetric, or severity-graded forms prior to adopting simplified thresholds.

Future refinement of NPC N3 staging should move beyond simply substituting one cutoff for another. Replacing a 6 cm threshold with 4 cm, the cricoid plane with an alternative anatomical landmark, or a diameter-based threshold with a volumetric one may perpetuate similar methodological limitations in altered forms. A more methodologically robust approach involves preserving the practical strengths of TNM staging, while periodically benchmarking existing criteria against integrated imaging–biological models that incorporate nodal burden, spatial extent, invasive behavior, EBV DNA, and selected host-related factors as these become standardized and clinically actionable.

In summary, current N3 criteria continue to serve as valuable pragmatic anchors for staging; however, their precise thresholds should be considered incompletely validated rather than biologically definitive. Future refinement of N3 classification should be informed by continuous risk characterization, standardized imaging definitions, interobserver reliability, external validation, and comprehensive assessment of clinical utility. This approach would enable NPC staging to maintain the simplicity necessary for global clinical implementation while more accurately reflecting the multidimensional and biologically heterogeneous nature of nodal risk.

## Figures and Tables

**Table 1:** Key N3-Related Descriptors in Nasopharyngeal Carcinoma: Rationale, Limitations, and Validation Needs.

Descriptor	Role in N3 Refinement	Risk Domain Captured	Key Limitation	Priority for Future Validation
>6 cm nodal-size threshold	Current AJCC/UICC N3 criterion	Large nodal size; historical bulky-disease marker	Historically inherited, unidimensional, and potentially discordant with true three-dimensional tumor burden	Model nodal diameter continuously; compare 6 cm with MRI-based diameter, nodal volume, and integrated burden metrics
Extension below the caudal border of the cricoid cartilage	Current AJCC/UICC N3 criterion	Inferior nodal extent/lower-neck involvement	Reproducible anatomical plane, but not a proven biological boundary; converts spatial spread into a binary rule	Evaluate inferior nodal extent as ordinal or spatially continuous; compare with nodal level, distance-based descriptors, and region-specific volume
Advanced radiologic extranodal extension	Current TNM-9 N3 criterion	Invasive nodal behavior beyond capsule	Requires standardized MRI definitions; potential interobserver variability; overlaps with necrosis, matting, and nodal volume	Test interobserver reliability, severity grading, external validation, and incremental value beyond nodal burden and EBV DNA
MRI-based maximal axial nodal diameter	Proposed imaging-derived size descriptor	MRI-defined nodal size	More imaging-relevant than palpation-era greatest dimension but still unidimensional	Characterize continuous diameter–risk relationship; validate proposed cutoffs externally
Nodal volume/nodal tumor volume	Proposed imaging-derived burden descriptor	Three-dimensional nodal tumor burden	Requires segmentation; sensitive to imaging protocol, contouring rules, necrosis, and matted nodes	Standardize segmentation; assess reproducibility; compare volume thresholds with continuous-volume models and current N categories
Middle-neck involvement/inferior spatial extent	Emerging spatial-risk descriptor	Graded inferior nodal dissemination above or around the cricoid plane	Recently proposed; not yet established as a staging criterion; anatomical definitions require validation	Validate across independent cohorts; compare with the cricoid-border rule and continuous/ordinal inferior-extent models
EBV DNA and integrated imaging–biological risk	Biological complement to anatomical staging	Systemic viral/tumor burden and multidimensional risk	Assay standardization, interlaboratory variability, and risk of model complexity	Integrate with imaging-derived nodal descriptors; assess calibration, decision utility, external validation, and implementability

Abbreviations: AJCC, American Joint Committee on Cancer; EBV, Epstein–Barr virus; MRI, magnetic resonance imaging; NPC, nasopharyngeal carcinoma; rENE, radiologic extranodal extension; TNM, tumor–node–metastasis; UICC, Union for International Cancer Control. Note: The table distinguishes prognostic-variable validity from threshold validity. A descriptor may be prognostically informative without its categorical cutoff or anatomical boundary being biologically or statistically optimal. “Current” indicates a descriptor incorporated into AJCC/UICC N staging, whereas “proposed” or “emerging” indicates descriptors discussed in the literature as candidates for future refinement rather than established staging criteria.

**Table 2:** Methodological Standards for Future Refinement of N3 Criteria in Nasopharyngeal Carcinoma.

Validation Step	Purpose	Recommended Approach	Key Output
Define the candidate descriptor	Ensure that the variable being evaluated is clinically and anatomically explicit	Specify whether the descriptor represents nodal diameter, nodal volume, inferior nodal extent, rENE severity, EBV DNA, or an integrated imaging–biological construct	Clearly defined descriptor suitable for reproducible measurement
Preserve the native variable scale	Avoid premature dichotomization and information loss	Model continuous variables as continuous, spatial descriptors as ordinal or distance-based, and rENE as severity-graded where possible	Full characterization of the underlying risk information
Characterize the risk relationship	Determine whether risk is linear, nonlinear, gradual, or threshold-like	Use restricted cubic splines, fractional polynomials, penalized spline-based Cox models, or comparable flexible approaches	Risk curve, nonlinearity assessment, and identification of potential inflection zones
Evaluate candidate thresholds only after risk-shape assessment	Prevent arbitrary or purely data-driven cutoff selection	Explore thresholds only if supported by the empirical risk curve, clinical rationale, or reproducible inflection pattern	Candidate threshold with biological and statistical rationale
Assess internal validity and stability	Estimate optimism and reduce overfitting	Apply bootstrap validation, cross-validation, shrinkage methods, and sensitivity analyses across candidate models	Optimism-corrected performance and model stability
Perform external validation	Determine generalizability across populations and practice settings	Validate in independent cohorts differing by endemicity, imaging protocol, treatment era, EBV DNA availability, systemic therapy, and follow-up	Evidence of transportability and robustness
Compare categorical and continuous representations	Determine whether simplification improves usability without excessive information loss	Compare cutoff-based models with continuous, ordinal, volumetric, and integrated models	Evidence that categorization is justified or that continuous modeling is preferable
Evaluate model performance comprehensively	Avoid reliance on *p*-values alone	Assess discrimination, calibration, Brier score, likelihood-based measures, time-dependent AUC, and reclassification where appropriate	Quantified statistical performance and clinical interpretability
Assess clinical utility	Determine whether the descriptor improves decision-making	Use decision-curve analysis, risk-threshold evaluation, and treatment-stratification scenarios	Net benefit and potential actionability
Test measurement reproducibility	Ensure feasibility for global staging use	Report interobserver agreement, segmentation reproducibility, imaging protocol requirements, and structured reporting standards	Evidence that the descriptor can be applied consistently
Evaluate incremental value beyond existing staging	Confirm that the candidate descriptor adds meaningful information	Test added value beyond current N category, T category, nodal volume, inferior extent, rENE, EBV DNA, and treatment-era variables	Independent and incremental prognostic contribution
Assess implementability	Balance prognostic precision with TNM simplicity	Consider required imaging resources, segmentation workload, assay standardization, reporting complexity, and global accessibility	Practical pathway for staging integration or use in prognostic models

Abbreviations: AUC, area under the curve; EBV, Epstein–Barr virus; MRI, magnetic resonance imaging; NPC, nasopharyngeal carcinoma; rENE, radiologic extranodal extension; TNM, tumor–node–metastasis. Note: This table outlines a validation pathway for candidate N3 refinements. The goal is not only to determine whether a descriptor is prognostically associated with outcome, but also to establish whether its proposed threshold or category is reproducible, biologically plausible, statistically robust, externally validated, clinically useful, and feasible for implementation in staging practice.

## Data Availability

No new data were created or analyzed in this study. Data sharing is not applicable to this article.
